# *catena*-Poly[[tetra­kis­(μ-penta­fluoro­benzoato-κ^2^ *O*:*O*′)dimolybdenum(II)]-μ-4,4′-bipyridine-κ^2^ *N*:*N*′]

**DOI:** 10.1107/S1600536811031734

**Published:** 2011-08-17

**Authors:** Li-Juan Han

**Affiliations:** a Department of Chemistry, Tongji University, Shanghai 200092, People’s Republic of China

## Abstract

In the title compound, [Mo_2_(C_7_F_5_O_2_)_4_(C_10_H_8_N_2_)]_*n*_, the mol­ecule forms a paddle-wheel-type structure. Each Mo_2_^4+^ unit is equatorially coordinated by four pentafluoro­benzoate groups, while the axial positions are occupied by two 4,4′-bipyridine mol­ecules. The Mo—Mo bond length of 2.1227 (4) Å is representative of a dimolybdenum quadruple bond. An infinite linear chain parallel to [110] is formed by the Mo_2_^4+^ unit coordinating axially to the two N atoms of the 4,4′-bipyridine ligand [Mo—N = 2.594 (2) Å]. The crystal packing shows mol­ecules linked together into a three-dimensional network *via* Mo—N coordination inter­actions and weak π–π stacking inter­actions between perfluoro­phenyl rings [centroid–centroid distance = 3.7280 (3) Å and centroid-to-plane distance = 3.6103 (12) Å between two penta­fluoro­phenyl rings].

## Related literature

For background to coordination polymers, see: Batten (2002 ▶); Kumar *et al.* (2004 ▶). For torsion angles about the penta­fluoro­benzoate anion, see: Reddy *et al.* (2004 ▶); Bach *et al.* (2001 ▶); For Mo–Mo quadruple bond lengths, see: Cotton *et al.* (2005 ▶).
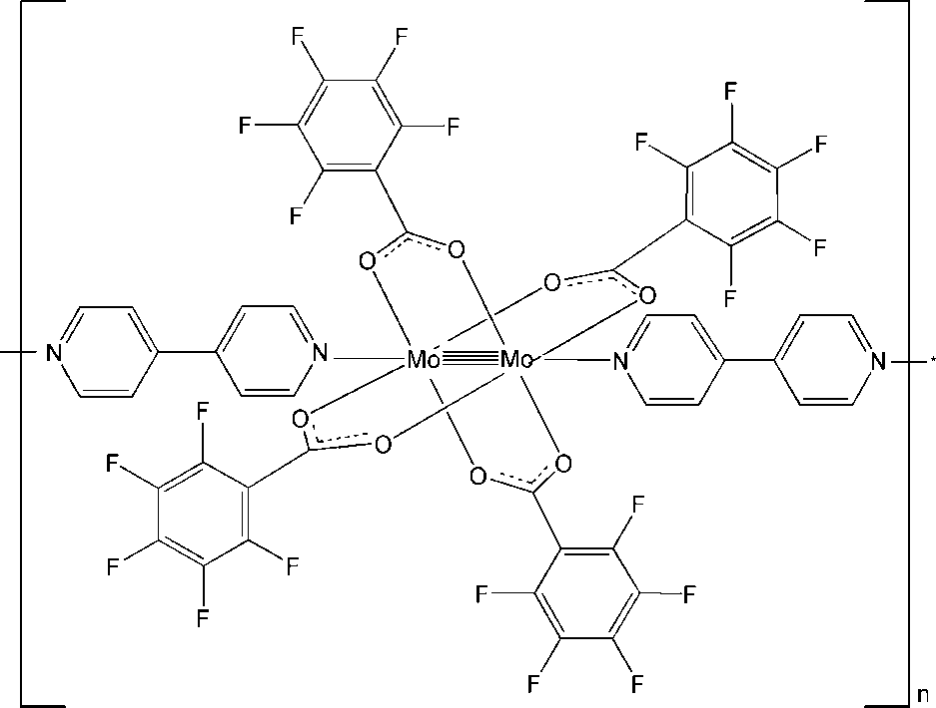


## Experimental

### 

#### Crystal data

[Mo_2_(C_7_F_5_O_2_)_4_(C_10_H_8_N_2_)]
*M* *_r_* = 1192.34
Triclinic, 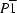

*a* = 8.8858 (8) Å
*b* = 9.9311 (9) Å
*c* = 11.1978 (10) Å
α = 101.158 (1)°
β = 94.697 (1)°
γ = 99.092 (1)°
*V* = 950.83 (15) Å^3^
*Z* = 1
Mo *K*α radiation
μ = 0.82 mm^−1^
*T* = 293 K
0.20 × 0.18 × 0.15 mm

#### Data collection

Bruker APEXII CCD diffractometer
Absorption correction: multi-scan (*SADABS*; Sheldrick, 2004 ▶) *T*_min_ = 0.849, *T*_max_ = 0.884
4937 measured reflections
3294 independent reflections
3072 reflections with *I* > 2σ(*I*)
*R*_int_ = 0.013

#### Refinement

*R*[*F* ^2^ > 2σ(*F* ^2^)] = 0.024
*wR*(*F* ^2^) = 0.061
*S* = 1.01
3294 reflections
316 parameters
H-atom parameters constrained
Δρ_max_ = 0.32 e Å^−3^
Δρ_min_ = −0.47 e Å^−3^

### 

Data collection: *APEX2* (Bruker, 2004 ▶); cell refinement: *SAINT-Plus* (Bruker, 2001 ▶); data reduction: *SAINT-Plus*; program(s) used to solve structure: *SHELXS97* (Sheldrick, 2008 ▶); program(s) used to refine structure: *SHELXL97* (Sheldrick, 2008 ▶); molecular graphics: *XP* in *SHELXTL* (Sheldrick, 2008 ▶); software used to prepare material for publication: *SHELXL97*.

## Supplementary Material

Crystal structure: contains datablock(s) global, I. DOI: 10.1107/S1600536811031734/jj2094sup1.cif

Structure factors: contains datablock(s) I. DOI: 10.1107/S1600536811031734/jj2094Isup2.hkl

Additional supplementary materials:  crystallographic information; 3D view; checkCIF report

## Figures and Tables

**Table 1:** Selected bond lengths (Å)

Mo1—O1	2.1124 (17)
Mo1—O6	2.1155 (16)
Mo1—O5	2.1427 (16)

**Table 2:** Hydrogen-bond geometry (Å, °)

*D*—H⋯*A*	*D*—H	H⋯*A*	*D*⋯*A*	*D*—H⋯*A*
C55—H55*A*⋯F35	0.93	2.55	3.152 (2)	122
C51—H51*A*⋯F33^ii^	0.93	2.78	2.987 (3)	94

Symmetry code: (ii) 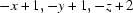.

**Fig. 1.**
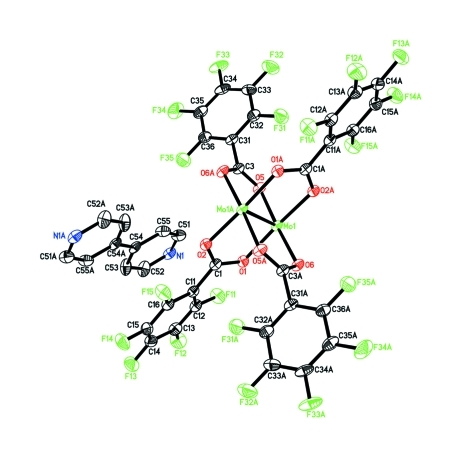
Molecular structure of the title compound drawn with displacement ellipsoids at the 30% probability level. All hydrogen atoms have been omitted for clarity.

**Fig. 2.**
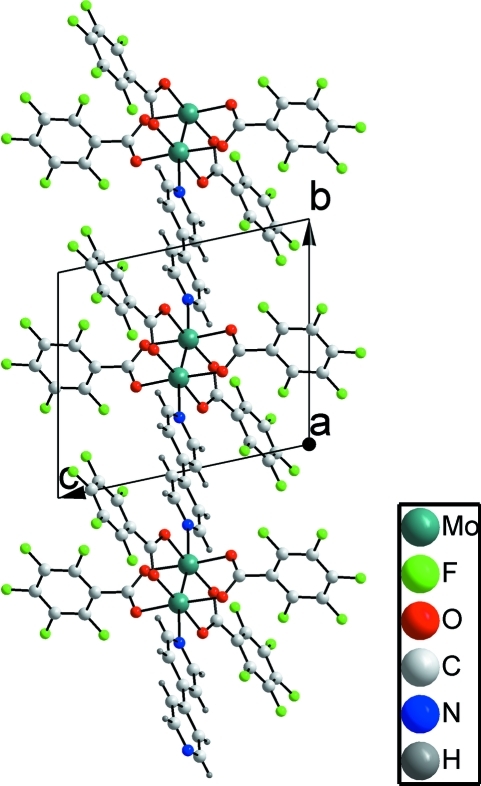
Part of a one-dimensional linear chain formed by a Mo–N coordination bond viewed along the a axis.

**Fig. 3.**
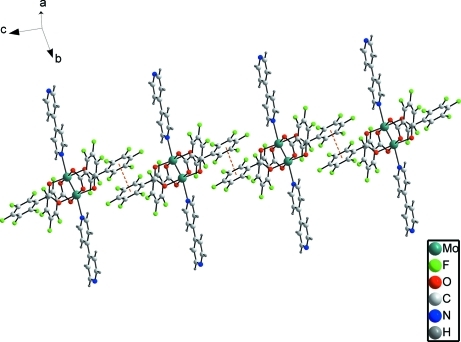
Part of a one-dimensional linear chain formed by the π–π intermolecular stacking interactions.

**Fig. 4.**
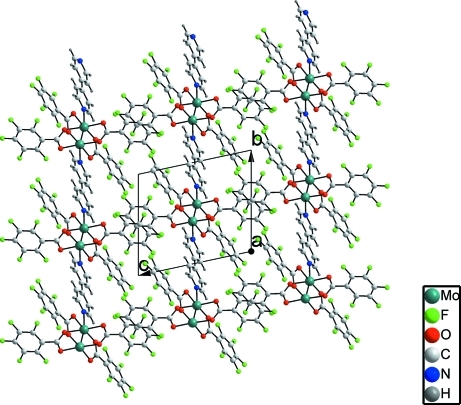
Molecular packing diagram of (I) with a view along the a axis.

## supplementary crystallographic information

### Comment

The design and construction of coordination polymers are of great interest due to their structural topologies and potential application as functional materials (Batten, 2002; Kumar, *et al.*, 2004). Here, we report the synthesis and crystal structure of the coordination polymer [Mo_2_(OOCC_6_F_5_)_4_(C_10_H_8_N_2_)]_n_.

In the title compound, Mo_2_(C_7_F_5_O_2_)_4_(C_10_H_8_N_2_), (I), the molecule forms a paddle-wheel-type structure. Each quadruply bonded Mo_2_^4+^ unit is equatorially coordinated by four pertafluoro-benzoate (OOCC_6_F_5_) groups and the axial positions have associated with them two 4, 4-bipyridine molecules (Fig. 1). The Mo–Mo bond length of 2.1227 (4) Å is representative for dimolybdenum quadruple bonds (Cotton *et al.*, 2005). The torsion angles between the C_6_F_5_ group and the connected chelating ring (Mo_2_OCO) are 72.081 (3)°, 75.537 (3)°, 22.059 (3)° and 22.422 (3)°, respectively, and relate to the O···F repulsion within the pentafluoro-benzonate anion (Reddy *et al.*, 2004; Bach *et al.*, 2001). Weak π–π stacking interactions between perfluorophenyl rings also affect the 72.081 (3)° and 75.537 (3)° torsion angles.

An infinite, linear chain coordination polymer is formed by the Mo_2_^4+^ unit coordinating axially to the two N atoms of the 4, 4-bipyridine ligand [Mo–N distance = 2.5938 (2) Å] (Fig. 2). A one-dimensional linear chain is generated by the π-π stacking between perfluorophenyl rings as viewed along the equatorial position of the Mo–Mo quadruple bonds (Fig. 3), [the center-to-center distance = 3.7280 (3) Å and center-to-plane distance = 3.6103 (0) Å between two pentafluorophenyl rings]. Crystal packing shows molecules linked together into a three-dimensional network (Fig. 4) *via* Mo–N coordination interactions and perfluorophenyl rings via weak π–π stacking interactions. Weak C–H···F intermolecular interactions further stabilize the crystal structure [(F···H distances = 2.7879 (15) Å].

### Experimental

4, 4-bipyridine (0.312 g, 2 mmol) was dissolved in dichloromethane (30 ml), and the solution was filtered to a Schelenk tube. Mo_2_(OOCC_6_F_5_)_4_ (0.207 g, 0.2 mmol) was dissolved in ethanol (10 ml), resulting in a clear yellow solution. The yellow solution was carefully layered on the top of the Schelenk tube. Solution diffusion at low temperature (in refrigetator) afforded yellow X-ray quality crystals after three days. Yield: 0.190 g (80%). Anal. Calcd. for C_38_H_8_N_2_O_8_F_20_Mo_2_: C, 38.28; H, 0.68; N, 2.35. Found: C, 38.13; H, 0.53; N, 2.37.

### Refinement

The H atoms were positioned geometrically and refined using the riding model with C–H = 0.93 Å for aromatic H, 0.96 Å for methyl H atoms. The *U*_iso_ parameters for H atoms were constrained to be 1.5*U*_eq_ of the carrier atom for the methyl H atoms and 1.2*U*_eq_ of the carrier atom for the remaining H atoms.

### Crystal data

**Table:**

[Mo_2_(C_7_F_5_O_2_)_4_(C_10_H_8_N_2_)]	*Z* = 1
*M**_r_* = 1192.34	*F*(000) = 578
Triclinic, *P*1	*D*_x_ = 2.082 Mg m^−^^3^
Hall symbol: -P 1	Mo *K*α radiation, λ = 0.71073 Å
*a* = 8.8858 (8) Å	Cell parameters from 4122 reflections
*b* = 9.9311 (9) Å	θ = 2.5–27.6°
*c* = 11.1978 (10) Å	µ = 0.82 mm^−^^1^
α = 101.158 (1)°	*T* = 293 K
β = 94.697 (1)°	Block, yellow
γ = 99.092 (1)°	0.20 × 0.18 × 0.15 mm
*V* = 950.83 (15) Å^3^	

### Data collection

**Table:**

Bruker APEXII CCD diffractometer	3294 independent reflections
Radiation source: fine-focus sealed tube	3072 reflections with *I* > 2σ(*I*)
graphite	*R*_int_ = 0.013
ω scans	θ_max_ = 25.0°, θ_min_ = 2.1°
Absorption correction: multi-scan (*SADABS*; Sheldrick, 2004)	*h* = −8→10
*T*_min_ = 0.849, *T*_max_ = 0.884	*k* = −11→11
4937 measured reflections	*l* = −13→12

### Refinement

**Table:**

Refinement on *F*^2^	Primary atom site location: structure-invariant direct methods
Least-squares matrix: full	Secondary atom site location: difference Fourier map
*R*[*F*^2^ > 2σ(*F*^2^)] = 0.024	Hydrogen site location: inferred from neighbouring sites
*wR*(*F*^2^) = 0.061	H-atom parameters constrained
*S* = 1.01	*w* = 1/[σ^2^(*F*_o_^2^) + (0.0323*P*)^2^ + 0.6373*P*] where *P* = (*F*_o_^2^ + 2*F*_c_^2^)/3
3294 reflections	(Δ/σ)_max_ = 0.001
316 parameters	Δρ_max_ = 0.32 e Å^−^^3^
0 restraints	Δρ_min_ = −0.47 e Å^−^^3^

### Special details

**Table:**

Geometry. All e.s.d.'s (except the e.s.d. in the dihedral angle between two l.s. planes) are estimated using the full covariance matrix. The cell e.s.d.'s are taken into account individually in the estimation of e.s.d.'s in distances, angles and torsion angles; correlations between e.s.d.'s in cell parameters are only used when they are defined by crystal symmetry. An approximate (isotropic) treatment of cell e.s.d.'s is used for estimating e.s.d.'s involving l.s. planes.
Refinement. Refinement of *F*^2^ against ALL reflections. The weighted *R*-factor *wR* and goodness of fit *S* are based on *F*^2^, conventional *R*-factors *R* are based on *F*, with *F* set to zero for negative *F*^2^. The threshold expression of *F*^2^ > σ(*F*^2^) is used only for calculating *R*-factors(gt) *etc*. and is not relevant to the choice of reflections for refinement. *R*-factors based on *F*^2^ are statistically about twice as large as those based on *F*, and *R*- factors based on ALL data will be even larger.

### Fractional atomic coordinates and isotropic or equivalent isotropic displacement parameters (Å^2^)

**Table:**

	*x*	*y*	*z*	*U*_iso_*/*U*_eq_	
Mo1	0.94356 (2)	0.58128 (2)	0.480838 (17)	0.02757 (8)	
F11	0.46291 (19)	0.34697 (17)	0.29542 (17)	0.0569 (4)	
F12	0.2509 (2)	0.1493 (2)	0.1523 (2)	0.0712 (5)	
F13	0.3174 (2)	−0.10294 (19)	0.05942 (18)	0.0682 (5)	
F14	0.6015 (2)	−0.15799 (17)	0.11139 (18)	0.0662 (5)	
F15	0.81465 (19)	0.03236 (17)	0.25713 (17)	0.0568 (4)	
F31	1.0522 (2)	0.7439 (2)	0.89717 (17)	0.0701 (5)	
F32	0.9848 (3)	0.8073 (2)	1.13037 (18)	0.0825 (6)	
F33	0.7539 (2)	0.6435 (2)	1.20533 (14)	0.0708 (6)	
F34	0.5986 (3)	0.4110 (2)	1.05142 (19)	0.0823 (6)	
F35	0.6643 (2)	0.3488 (2)	0.81764 (17)	0.0770 (6)	
O1	0.75702 (18)	0.43333 (16)	0.38004 (15)	0.0332 (4)	
O2	0.87528 (19)	0.26377 (17)	0.42436 (15)	0.0345 (4)	
O5	0.85431 (19)	0.59500 (18)	0.65400 (15)	0.0355 (4)	
O6	1.03083 (19)	0.58024 (17)	0.31053 (15)	0.0352 (4)	
C1	0.7665 (3)	0.3051 (3)	0.3681 (2)	0.0330 (5)	
C3	0.8955 (3)	0.5167 (3)	0.7219 (2)	0.0325 (5)	
C11	0.6483 (3)	0.1984 (3)	0.2857 (2)	0.0341 (5)	
C12	0.5018 (3)	0.2234 (3)	0.2528 (2)	0.0391 (6)	
C13	0.3913 (3)	0.1235 (3)	0.1796 (3)	0.0456 (7)	
C14	0.4231 (3)	−0.0065 (3)	0.1325 (3)	0.0467 (7)	
C15	0.5677 (3)	−0.0340 (3)	0.1609 (2)	0.0437 (6)	
C16	0.6762 (3)	0.0656 (3)	0.2357 (2)	0.0382 (6)	
C31	0.8594 (3)	0.5460 (3)	0.8527 (2)	0.0346 (5)	
C32	0.9392 (3)	0.6610 (3)	0.9340 (2)	0.0428 (6)	
C33	0.9070 (4)	0.6945 (3)	1.0538 (2)	0.0497 (7)	
C34	0.7909 (3)	0.6100 (3)	1.0917 (2)	0.0480 (7)	
C35	0.7115 (3)	0.4929 (3)	1.0133 (3)	0.0502 (7)	
C36	0.7458 (3)	0.4611 (3)	0.8941 (2)	0.0437 (6)	
N1	0.2434 (3)	0.2426 (2)	0.5169 (2)	0.0434 (5)	
C51	0.3883 (4)	0.2800 (3)	0.5640 (2)	0.0498 (7)	
H51A	0.4226	0.3727	0.6027	0.060*	
C52	0.1981 (3)	0.1097 (3)	0.4617 (3)	0.0555 (8)	
H52A	0.0966	0.0810	0.4270	0.067*	
C53	0.2934 (3)	0.0113 (3)	0.4527 (3)	0.0540 (8)	
H53A	0.2554	−0.0805	0.4130	0.065*	
C54	0.4463 (3)	0.0507 (3)	0.5034 (2)	0.0377 (6)	
C55	0.4927 (3)	0.1900 (3)	0.5594 (2)	0.0467 (7)	
H55A	0.5938	0.2230	0.5938	0.056*	

### Atomic displacement parameters (Å^2^)

**Table:**

	*U*^11^	*U*^22^	*U*^33^	*U*^12^	*U*^13^	*U*^23^
Mo1	0.03074 (12)	0.03149 (12)	0.02725 (12)	0.01909 (8)	0.00822 (8)	0.00964 (8)
F11	0.0419 (9)	0.0543 (10)	0.0761 (12)	0.0260 (8)	0.0033 (8)	0.0049 (9)
F12	0.0397 (9)	0.0805 (13)	0.0925 (14)	0.0211 (9)	−0.0114 (9)	0.0154 (11)
F13	0.0585 (11)	0.0614 (11)	0.0735 (12)	−0.0033 (9)	−0.0153 (10)	0.0079 (9)
F14	0.0698 (12)	0.0416 (9)	0.0795 (13)	0.0163 (9)	−0.0058 (10)	−0.0049 (9)
F15	0.0458 (9)	0.0479 (9)	0.0739 (12)	0.0251 (8)	−0.0035 (8)	−0.0034 (8)
F31	0.0668 (12)	0.0753 (13)	0.0585 (11)	−0.0139 (10)	0.0155 (9)	0.0085 (9)
F32	0.1018 (17)	0.0801 (14)	0.0504 (11)	0.0059 (12)	0.0050 (11)	−0.0135 (10)
F33	0.0872 (14)	0.1141 (16)	0.0306 (8)	0.0598 (12)	0.0245 (9)	0.0213 (9)
F34	0.0797 (14)	0.1097 (17)	0.0669 (12)	0.0041 (12)	0.0374 (11)	0.0393 (12)
F35	0.0870 (15)	0.0773 (13)	0.0534 (11)	−0.0205 (11)	0.0149 (10)	0.0069 (10)
O1	0.0319 (9)	0.0335 (9)	0.0390 (9)	0.0169 (7)	0.0043 (7)	0.0098 (7)
O2	0.0357 (9)	0.0349 (9)	0.0375 (9)	0.0162 (7)	0.0044 (7)	0.0108 (7)
O5	0.0391 (9)	0.0449 (10)	0.0318 (9)	0.0260 (8)	0.0126 (7)	0.0124 (7)
O6	0.0403 (10)	0.0412 (9)	0.0334 (9)	0.0225 (8)	0.0121 (7)	0.0153 (7)
C1	0.0331 (13)	0.0395 (13)	0.0328 (12)	0.0160 (11)	0.0105 (10)	0.0123 (10)
C3	0.0317 (12)	0.0401 (13)	0.0312 (12)	0.0150 (11)	0.0092 (10)	0.0116 (10)
C11	0.0344 (13)	0.0394 (13)	0.0339 (12)	0.0131 (11)	0.0097 (10)	0.0131 (10)
C12	0.0380 (14)	0.0440 (14)	0.0426 (14)	0.0178 (12)	0.0109 (11)	0.0162 (12)
C13	0.0342 (14)	0.0573 (17)	0.0504 (16)	0.0141 (13)	0.0022 (12)	0.0196 (14)
C14	0.0457 (16)	0.0500 (16)	0.0420 (15)	0.0011 (13)	−0.0020 (13)	0.0126 (13)
C15	0.0505 (16)	0.0375 (14)	0.0441 (15)	0.0117 (12)	0.0056 (13)	0.0079 (12)
C16	0.0351 (13)	0.0423 (14)	0.0419 (14)	0.0151 (11)	0.0053 (11)	0.0132 (11)
C31	0.0365 (13)	0.0452 (14)	0.0298 (12)	0.0206 (11)	0.0109 (10)	0.0128 (11)
C32	0.0408 (15)	0.0538 (16)	0.0387 (14)	0.0140 (13)	0.0112 (12)	0.0141 (12)
C33	0.0576 (18)	0.0579 (17)	0.0334 (14)	0.0234 (15)	0.0003 (13)	0.0008 (13)
C34	0.0540 (17)	0.076 (2)	0.0283 (13)	0.0404 (16)	0.0159 (12)	0.0177 (14)
C35	0.0463 (16)	0.073 (2)	0.0427 (16)	0.0201 (15)	0.0188 (13)	0.0262 (15)
C36	0.0443 (15)	0.0543 (16)	0.0357 (14)	0.0124 (13)	0.0101 (12)	0.0117 (12)
N1	0.0503 (14)	0.0533 (14)	0.0405 (12)	0.0343 (11)	0.0176 (11)	0.0187 (10)
C51	0.0609 (19)	0.0551 (17)	0.0396 (15)	0.0382 (15)	0.0052 (13)	0.0025 (13)
C52	0.0399 (16)	0.0552 (18)	0.084 (2)	0.0259 (14)	0.0165 (15)	0.0281 (17)
C53	0.0416 (16)	0.0402 (15)	0.090 (2)	0.0202 (13)	0.0163 (15)	0.0234 (15)
C54	0.0446 (15)	0.0458 (14)	0.0363 (13)	0.0264 (12)	0.0179 (11)	0.0211 (11)
C55	0.0491 (16)	0.0586 (17)	0.0360 (14)	0.0320 (14)	0.0019 (12)	0.0018 (12)

### Geometric parameters (Å, °)

**Table:**

Mo1—O2^i^	2.0955 (17)	C11—C12	1.397 (3)
Mo1—O1	2.1124 (17)	C11—C16	1.398 (3)
Mo1—O6	2.1155 (16)	C12—C13	1.366 (4)
Mo1—Mo1^i^	2.1227 (4)	C13—C14	1.379 (4)
Mo1—O5	2.1427 (16)	C14—C15	1.379 (4)
Mo1—N1^ii^	2.594 (2)	C15—C16	1.362 (4)
F11—C12	1.337 (3)	C31—C32	1.368 (4)
F12—C13	1.335 (3)	C31—C36	1.379 (4)
F13—C14	1.325 (3)	C32—C33	1.383 (4)
F14—C15	1.340 (3)	C33—C34	1.373 (4)
F15—C16	1.338 (3)	C34—C35	1.365 (4)
F31—C32	1.338 (3)	C35—C36	1.381 (4)
F32—C33	1.325 (3)	N1—C51	1.318 (4)
F33—C34	1.333 (3)	N1—C52	1.327 (4)
F34—C35	1.341 (3)	C51—C55	1.383 (4)
F35—C36	1.333 (3)	C51—H51A	0.9300
O1—C1	1.272 (3)	C52—C53	1.385 (4)
O2—C1	1.274 (3)	C52—H52A	0.9300
O2—Mo1^i^	2.0955 (17)	C53—C54	1.393 (4)
O5—C3	1.261 (3)	C53—H53A	0.9300
O6—C3^i^	1.261 (3)	C54—C55	1.385 (4)
C1—C11	1.480 (3)	C54—C54^iii^	1.489 (5)
C3—O6^i^	1.261 (3)	C55—H55A	0.9300
C3—C31	1.508 (3)		
			
O2^i^—Mo1—O1	176.82 (6)	F15—C16—C11	120.6 (2)
O2^i^—Mo1—O6	92.90 (7)	C15—C16—C11	122.5 (2)
O1—Mo1—O6	85.79 (7)	C32—C31—C36	118.0 (2)
O2^i^—Mo1—Mo1^i^	92.41 (4)	C32—C31—C3	119.8 (2)
O1—Mo1—Mo1^i^	90.57 (4)	C36—C31—C3	122.2 (2)
O6—Mo1—Mo1^i^	93.70 (4)	F31—C32—C31	119.4 (2)
O2^i^—Mo1—O5	85.43 (7)	F31—C32—C33	118.7 (3)
O1—Mo1—O5	95.73 (7)	C31—C32—C33	121.9 (3)
O6—Mo1—O5	176.63 (6)	F32—C33—C34	120.4 (3)
Mo1^i^—Mo1—O5	89.29 (4)	F32—C33—C32	120.8 (3)
C1—O1—Mo1	117.52 (15)	C34—C33—C32	118.9 (3)
C1—O2—Mo1^i^	116.51 (15)	F33—C34—C35	119.8 (3)
C3—O5—Mo1	117.24 (14)	F33—C34—C33	119.8 (3)
C3^i^—O6—Mo1	114.32 (14)	C35—C34—C33	120.5 (2)
O1—C1—O2	122.5 (2)	F34—C35—C34	119.9 (3)
O1—C1—C11	119.5 (2)	F34—C35—C36	120.3 (3)
O2—C1—C11	118.0 (2)	C34—C35—C36	119.8 (3)
O5—C3—O6^i^	124.7 (2)	F35—C36—C31	119.9 (2)
O5—C3—C31	117.2 (2)	F35—C36—C35	119.0 (3)
O6^i^—C3—C31	118.1 (2)	C31—C36—C35	121.0 (3)
C12—C11—C16	115.6 (2)	C51—N1—C52	116.5 (2)
C12—C11—C1	122.4 (2)	N1—C51—C55	124.1 (3)
C16—C11—C1	122.0 (2)	N1—C51—H51A	117.9
F11—C12—C13	117.1 (2)	C55—C51—H51A	117.9
F11—C12—C11	120.6 (2)	N1—C52—C53	123.8 (3)
C13—C12—C11	122.3 (2)	N1—C52—H52A	118.1
F12—C13—C12	121.0 (3)	C53—C52—H52A	118.1
F12—C13—C14	118.7 (3)	C52—C53—C54	119.7 (3)
C12—C13—C14	120.3 (2)	C52—C53—H53A	120.2
F13—C14—C15	120.5 (3)	C54—C53—H53A	120.2
F13—C14—C13	120.6 (3)	C55—C54—C53	116.0 (2)
C15—C14—C13	119.0 (3)	C55—C54—C54^iii^	122.0 (3)
F14—C15—C16	120.4 (2)	C53—C54—C54^iii^	122.0 (3)
F14—C15—C14	119.3 (3)	C51—C55—C54	119.9 (3)
C16—C15—C14	120.3 (2)	C51—C55—H55A	120.1
F15—C16—C15	116.8 (2)	C54—C55—H55A	120.1
			
O6—Mo1—O1—C1	91.22 (16)	C1—C11—C16—F15	−2.7 (4)
Mo1^i^—Mo1—O1—C1	−2.45 (16)	C12—C11—C16—C15	−0.8 (4)
O5—Mo1—O1—C1	−91.80 (16)	C1—C11—C16—C15	179.2 (2)
O2^i^—Mo1—O5—C3	−89.66 (18)	O5—C3—C31—C32	−72.1 (3)
O1—Mo1—O5—C3	93.32 (18)	O6^i^—C3—C31—C32	105.4 (3)
Mo1^i^—Mo1—O5—C3	2.82 (18)	O5—C3—C31—C36	107.0 (3)
O2^i^—Mo1—O6—C3^i^	98.99 (17)	O6^i^—C3—C31—C36	−75.6 (3)
O1—Mo1—O6—C3^i^	−83.91 (17)	C36—C31—C32—F31	178.7 (2)
Mo1^i^—Mo1—O6—C3^i^	6.38 (17)	C3—C31—C32—F31	−2.2 (4)
Mo1—O1—C1—O2	7.3 (3)	C36—C31—C32—C33	−1.2 (4)
Mo1—O1—C1—C11	−172.39 (15)	C3—C31—C32—C33	177.9 (2)
Mo1^i^—O2—C1—O1	−8.4 (3)	F31—C32—C33—F32	0.9 (4)
Mo1^i^—O2—C1—C11	171.27 (15)	C31—C32—C33—F32	−179.3 (3)
Mo1—O5—C3—O6^i^	−9.2 (3)	F31—C32—C33—C34	179.8 (2)
Mo1—O5—C3—C31	168.03 (16)	C31—C32—C33—C34	−0.3 (4)
O1—C1—C11—C12	−22.4 (3)	F32—C33—C34—F33	1.7 (4)
O2—C1—C11—C12	157.9 (2)	C32—C33—C34—F33	−177.3 (2)
O1—C1—C11—C16	157.6 (2)	F32—C33—C34—C35	−179.4 (3)
O2—C1—C11—C16	−22.1 (3)	C32—C33—C34—C35	1.7 (4)
C16—C11—C12—F11	−179.9 (2)	F33—C34—C35—F34	−1.1 (4)
C1—C11—C12—F11	0.0 (4)	C33—C34—C35—F34	179.9 (3)
C16—C11—C12—C13	2.0 (4)	F33—C34—C35—C36	177.4 (3)
C1—C11—C12—C13	−178.1 (2)	C33—C34—C35—C36	−1.5 (4)
F11—C12—C13—F12	0.0 (4)	C32—C31—C36—F35	179.4 (2)
C11—C12—C13—F12	178.2 (2)	C3—C31—C36—F35	0.3 (4)
F11—C12—C13—C14	−179.9 (2)	C32—C31—C36—C35	1.3 (4)
C11—C12—C13—C14	−1.8 (4)	C3—C31—C36—C35	−177.8 (2)
F12—C13—C14—F13	1.4 (4)	F34—C35—C36—F35	0.5 (4)
C12—C13—C14—F13	−178.6 (2)	C34—C35—C36—F35	−178.0 (3)
F12—C13—C14—C15	−179.6 (3)	F34—C35—C36—C31	178.6 (3)
C12—C13—C14—C15	0.3 (4)	C34—C35—C36—C31	0.0 (4)
F13—C14—C15—F14	1.7 (4)	C52—N1—C51—C55	−0.3 (4)
C13—C14—C15—F14	−177.2 (3)	C51—N1—C52—C53	0.6 (4)
F13—C14—C15—C16	179.7 (2)	N1—C52—C53—C54	−0.1 (5)
C13—C14—C15—C16	0.8 (4)	C52—C53—C54—C55	−0.7 (4)
F14—C15—C16—F15	−0.7 (4)	C52—C53—C54—C54^iii^	179.9 (3)
C14—C15—C16—F15	−178.7 (2)	N1—C51—C55—C54	−0.5 (4)
F14—C15—C16—C11	177.5 (2)	C53—C54—C55—C51	1.0 (4)
C14—C15—C16—C11	−0.5 (4)	C54^iii^—C54—C55—C51	−179.6 (3)
C12—C11—C16—F15	177.3 (2)		

Symmetry codes: (i) −*x*+2, −*y*+1, −*z*+1; (ii) −*x*+1, −*y*+1, −*z*+1; (iii) −*x*+1, −*y*, −*z*+1.

### Hydrogen-bond geometry (Å, °)

**Table:**

*D*—H···*A*	*D*—H	H···*A*	*D*···*A*	*D*—H···*A*
C55—H55A···F35	0.93	2.55	3.152 (2)	122.
C51—H51A···F33^iv^	0.93	2.78	2.987 (3)	94.

Symmetry codes:  (iv) −*x*+1, −*y*+1, −*z*+2.
